# Arming Oncolytic Adenoviruses: Effect of Insertion Site and Splice Acceptor on Transgene Expression and Viral Fitness

**DOI:** 10.3390/ijms21145158

**Published:** 2020-07-21

**Authors:** Martí Farrera-Sal, Jana de Sostoa, Estela Nuñez-Manchón, Rafael Moreno, Cristina Fillat, Miriam Bazan-Peregrino, Ramon Alemany

**Affiliations:** 1 ProCure Program, Institut Català d’Oncologia, and Oncobell Program IDIBELL, 08908 L’Hospitalet de Llobregat, Spain; marti.farrera@gmail.com (M.F.-S.); jana.sostoa@gmail.com (J.d.S.); rafamoreno@iconcologia.net (R.M.); 2 VCN Biosciences S.L., 08174 Sant Cugat, Spain; mbazan@vcnbiosciences.com; 3 Institut d’investigacions Biomèdiques August Pi i Sunyer (IDIBAPS), Centro de Investigación Biomédica en Red de Enfermedades Raras (CIBERER), Universitat de Barcelona, 08036 Barcelona, Spain; nunez2@clinic.cat (E.N.-M.); CFILLAT@clinic.cat (C.F.)

**Keywords:** oncolytic adenovirus, adenovirus, transgenes, splice acceptor, codon usage

## Abstract

Oncolytic adenoviruses (OAds) present limited efficacy in clinics. The insertion of therapeutic transgenes into OAds genomes, known as “arming OAds”, has been the main strategy to improve their therapeutic potential. Different approaches were published in the decade of the 2000s, but with few comparisons. Most armed OAds have complete or partial E3 deletions, leading to a shorter half-life in vivo. We generated E3+ OAds using two insertion sites, After-fiber and After-E4, and two different splice acceptors linked to the major late promoter, either the Ad5 protein IIIa acceptor (IIIaSA) or the Ad40 long fiber acceptor (40SA). The highest transgene levels were obtained with the After-fiber location and 40SA. However, the set of codons of the transgene affected viral fitness, highlighting the relevance of transgene codon usage when arming OAds using the major late promoter.

## 1. Introduction

Oncolytic adenoviruses (OAds) have been tested in clinical trials with a relatively safe profile but limited efficacy as monotherapy, with few complete and sustained tumor regressions [1,2]. In consequence, enhancing oncolytic potency is a crucial need in the field. To achieve this, therapeutic transgenes have been inserted in the OAd genome, also known as “arming OAds” [3]. Transgene-encoded proteins or RNAs (miRNA, shRNA, siRNA) are expressed to eliminate neighboring and distant uninfected cancer cells. Although the nature of the therapeutic transgene would be the first important feature of an armed OAd, the amount and timing of transgene expression are also pivotal.

Adenoviruses (Ads) maximize their genome coding capacity by generating early and late transcription units (active either before or after virus DNA replication, respectively). These units are controlled by multiple promoters, which in turn generate different RNAs and proteins by alternative splicing [4,5]. The adenovirus genome can only stably accommodate 2 kb of additional DNA beyond the size of the normal genome [6]. Therefore, gene deletions are needed to introduce larger transgenes. Early transcription unit 3 (E3) encodes for immune-inhibitory proteins non-essential for viral replication in vitro [7,8,9] and is deleted or replaced by insert transgenes. Based on this strategy, most armed OAds have partial or complete E3 deletions. However, due to the immune evasion functions of E3, E3-deleted viruses were reported to be cleared in vivo much more rapidly than wild-type viruses [10,11,12], suggesting that E3 deletion may contribute to the fast clearance of adenoviruses in patients. In this context, non-E3 deleted viruses are our focus of interest, despite the genome packaging limitation.

Different strategies to control transgene transcription have been developed. The most relevant in terms of transgene levels and selective expression are the use of internal ribosome entry sites (IRES) and splice acceptors (SAs). In head to head comparisons, IRES expressed higher transgene levels than SA, but at the expense of selectivity [13,14]. Nevertheless, in non-E3 deleted viruses, the use of shorter sequences such as SA (50 bp) compared to IRES (300–600 bp) could be imperative.

To our knowledge, four different splice acceptors have been used to insert transgenes into OAds: the SA from Ad5 protein IIIa (IIIaSA) [15,16], the SA from Ad40 long fiber (40SA) [17], the Ad41 long fiber (41SA) [14], and an artificial splice acceptor site derived from the *beta globulin* gene (BPSA) [18,19]. Nevertheless, no direct comparisons have been published for expression levels or virus replication compatibility. In this study, we compare transgene expression and virus replication between armed OAds with IIIaSA or 40SA.

Apart from the SA used to control transgene expression, the insertion site into the Ad genome is also critical. After viral DNA replication, Ad late genes are transcribed at higher levels compared to the early genes. Thus, linking the transgene expression to late gene transcription control leads to higher expression levels [20]. Two transgene positions have been reported in non-E3 deleted viruses: after the fiber gene (L5) as a new transcription unit, also named L6 [14,16], and also downstream of the 23K protease gene in L3 [20].

We compare the transgene levels and viral fitness of two different insertion sites (After-fiber and After-E4, previously only reported for E3-deleted viruses [18,19]) with two different splice acceptors (IIIaSA and 40SA). Expression and fitness were evaluated with a reporter luciferase gene and with two therapeutic transgenes: a bispecific T-cell engager (BiTE) against fibroblast activation protein (FAP, FBiTE) [21] and human hyaluronidase (PH20; *SPAM1)* [22].

## 2. Results

We previously published the generation of ICOVIR-15K (ICO15K), an E1a-Δ24-based oncolytic adenovirus with palindromic E2F binding sites in the *E1a promoter,* and an RGDK motif replacing the KKTK heparan sulfate glycosaminoglycan-binding domain in the fiber shaft [23] (Figure S1). This virus presented a favorable toxicity profile and increased tumor targeting in vivo. We used ICO15K as a platform to incorporate three transgenes: luciferase, FBiTE, or PH20. All transgenes were inserted in a cassette containing an upstream SA and Kozak sequence and a downstream polyA signal.

### 2.1. Generation and Characterization of Luciferase-Armed Oncolytic Adenoviruses

**Figure 1:**
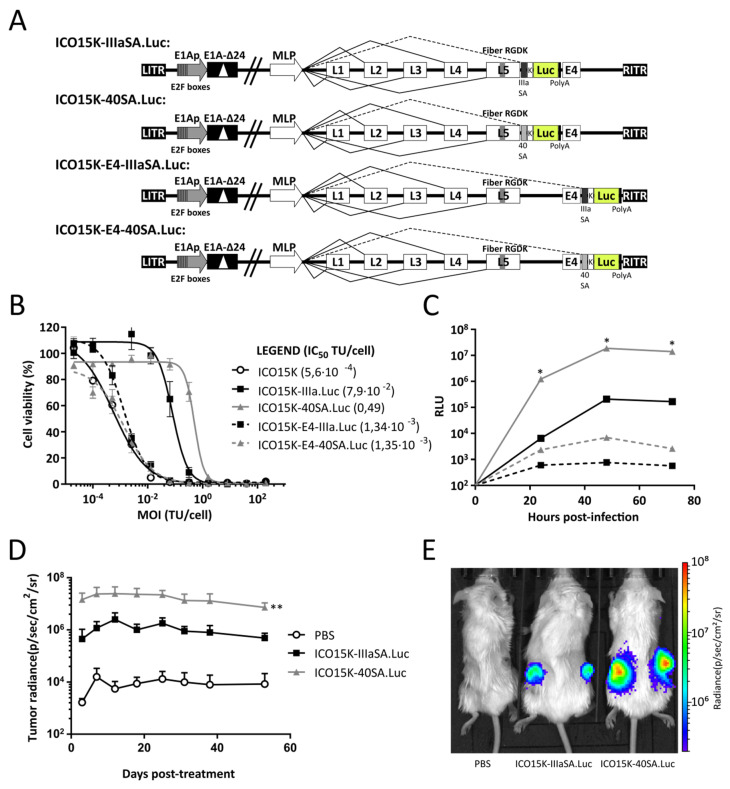
(**A**) Genomic schematic representation of luciferase-expressing OAds generated in this study. (**B**) Dose-dependent cytotoxic assay in A549 cells in vitro. (**C**) Luciferase assay in vitro. A549 cells were infected with five transfecting units (TU) per cell, and relative light units (RLU) were monitored 72 h post-infection. * *p* < 0.05 significant vs. ICO15K-E4-IIIa.Luc based on the Kruskal–Wallis test and Dunn’s *post hoc* test. (**D**) SCID/Beige mice bearing A549 tumors (n ≥ 10 tumors per group) were injected intratumorally with 10^9^ viral particles (vp), and tumor luminescence was measured 53 days post-treatment. ** *p* < 0.01 significant versus other groups based on two-way ANOVA and Tukey’s *post hoc* test. (**E**) Representative images of tumor luminescence in mice at day 12 post-treatment.

The genetic element to control transgene expression and the transgene insertion site critically determines the efficacy of the armed OAds approach. To easily quantify transgene levels, Click Beetle Green luciferase (Luc) controlled by either the splice acceptor IIIaSA or 40SA was inserted in two different genome positions: immediately downstream of the fiber gene (“After-fiber” location) or between the E4 transcription unit and the right ITR (“After-E4” location, labeled as “E4” in the virus name). Accordingly, four different viruses were generated and successfully rescued: ICO15K-IIIaSA.Luc, ICO15K-40SA.Luc, ICO15K-E4-IIIaSA.Luc, and ICO15K-E4–40SA.Luc (Figure 1A).

A dose-response cytotoxicity assay in the reference A549 cell line was performed to address the oncolytic potential of luciferase-expressing viruses. Each virus showed a dose-dependent oncolytic effect in vitro. However, comparing insertion sites, the inhibitory concentration (IC_50_) values were substantially higher (less cytotoxic potency) for After-fiber-armed OAds (IC_50_: 0.49 and 7.9 × 10^−2^) than for the parental ICO15K (IC_50_: 5.6 × 10^−4^) and for After-E4-armed viruses (IC_50_: 1.34 × 10^−3^ and 1.35 × 10^−3^) (Figure 1B). Regarding the splice acceptor, the virus armed with After-fiber with IIIaSA showed more cytotoxic potency than with 40SA. Conversely, similar IC_50_ were found for both After-E4 viruses.

### 2.2. After-Fiber-Armed Virus with 40SA Produced the Highest Luminescence Levels In Vitro and In Vivo

To test the magnitude and timing of the transgene expression, A549 cells were infected in vitro, and luciferase activity was monitored 72 h post-infection. The emitted relative light units (RLU) increased with time for all viruses. However, the After-fiber-armed viruses presented higher levels of expression compared to After-E4-armed OAds (Figure 1C). Comparing SAs, the 40SA produced more luciferase than the IIIaSA. The luciferase levels of ICO15K-40SA.Luc were 100-fold higher than the other viruses at different time points.

Despite the better oncolytic properties of After-E4-armed OAds in vitro, we discarded them for in vivo tests due to the lower transgene expression (2- to 4-log lower than After-fiber viruses). Consequently, we only considered the After-fiber viruses as candidates to test transgene kinetics in vivo. SCID/beige mice bearing A549 tumors were intratumorally injected and luminescence was monitored for 53 days. The virus with 40SA significantly emitted 20-fold more luminescence in tumors than that with IIIaSA (Figure 1D,E).

### 2.3. Generation and Characterization of FBiTE-Expressing Oncolytic Adenoviruses

**Figure 2:**
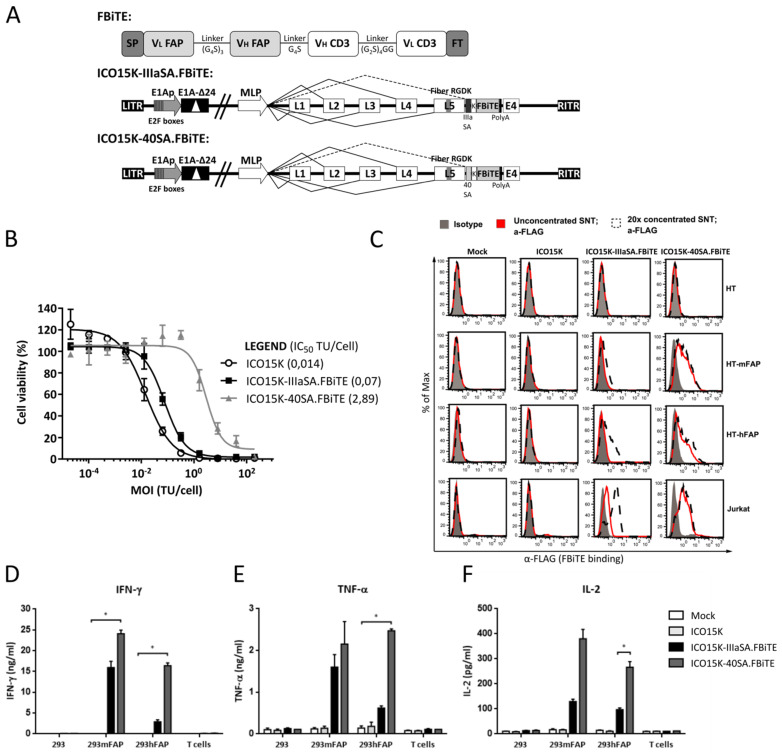
(**A**) Schematic representation of FAP-BiTE molecule (FBiTE) and FBiTE-armed OAds (SP: Signal peptide, FT: Flag-Tag). (**B**) Dose-dependent cytotoxic assay in A549 cells five days post-infection. (**C**) Binding assay. The presence of bound FBiTE molecules was assessed by flow cytometry against Flag-Tag in different target cells. (**D–F**) Average concentrations of IFN-ɣ (D), TNF-α (E) and IL-2 (F) cytokines were measured by ELISA assay using supernatants from 24h co-cultures of HEK293 (293), 293mFAP or 293hFAP cells with T cells (E:T=5) and indicated supernatants. Mean values ± SD are plotted (*n* = 3). * *p* < 0.05 versus Mock or ICO15K based on the Kruskal–Wallis test and Dunn’s *post hoc* test.

The results above indicated that the After-E4 insertion site produced low levels of transgene expression in E3+ (E3 not deleted) OAds. Thus, we discarded this insertion site and introduced therapeutic genes After-fiber for further splice acceptor comparisons. We recently published the generation of an OAd armed with an FAP-targeting BiTE (FBiTE) under IIIaSA After-fiber, ICO15K-IIIaSA.FBiTE [21]. The FBiTE molecule was engineered by joining with flexible GS linkers two single chains (ScFv), one specific for human CD3ε, and the other for murine and human FAP. In order to compare the mentioned SAs, we generated a new OAd harboring the FBiTE controlled by the 40SA, named ICO15K-40SA.FBiTE (Figure 2A).

The oncolytic properties of ICO15K-IIIaSA.FBiTE and ICO15K-40SA.FBiTE were assessed in vitro. ICO15K-IIIaSA.FBiTE (IC_50_ = 0.07) had a slightly reduced cytotoxic effect compared to the parental virus (IC_50_ = 0.014). However, the virus with the 40SA (IC_50_ = 2.89) presented significantly higher IC_50_ values than the parental and the IIIaSA viruses in the A549 cell line (Figure 2B). Therefore, as observed with luciferase, arming OAds using the 40SA reduced viral replication.

### 2.4. The After-Fiber-Armed Virus with 40SA Expressed Higher FBiTE Levels than IIIaSA

We next determined the amount of FBiTE produced and secreted. To this end, we generated supernatants from infected cells and tested them in binding assays. For the binding assay, HT-1080 (HT) cells were genetically modified to express human FAP (HT-hFAP) or murine FAP (HT-mFAP). Binding of FBiTE specifically to the modified cells was detected by flow cytometry using both ICO15K-IIIaSA.FBiTE and ICO15K-40SA.FBiTE supernatants. Moreover, FBiTE molecules were also able to bind to CD3-positive Jurkat cells, confirming the functionality of both ScFvs of the BiTE. Both FAP+ and CD3+ bindings were more pronounced using 40SA-armed virus supernatants, suggesting higher transgene production than with IIIaSA. These results were confirmed with concentrated supernatants (see Materials and methods 4.6). The 40SA supernatants saturated the binding level without the need of concentration. On the other hand, IIIaSA supernatants presented a considerable binding increase after the 20-fold concentration (Figure 2C).

Aiming to evaluate the functionality of FBiTE, we analyzed the T-cell cytokine production in co-cultures with 293 control cells, 293 murine-expressing FAP (293mFAP) or 293 human-expressing FAP cells (293hFAP), and the presence of supernatants from adenovirus-infected cells (ICO15K, ICO15K-IIIaSA.FBiTE or ICO15K-40SA.FBiTE) or uninfected cells (mock). Supernatants were obtained 24 h after co-culture, and T-cell activation was assessed by quantifying IFN-ɣ (Figure 2D), TNF-α (Figure 2E), and IL-2 (Figure 2F). Cytokine release was observed in co-cultures of T cells and FAP-expressing cells in the presence of ICO15K-IIIaSA.FBiTE and ICO15K-40SA.FBiTE supernatants. Cytokine levels were generally higher in the presence of murine FAP-expressing cells compared to human FAP-expressing cells. This can be explained by the FAP5 monoclonal antibody affinity from which the scFv in FBiTE is derived [24].

Importantly, there was no cytokine release in the presence of 293 control cells or when using supernatants from either ICO15K or mock-infections. Again, the T-cell cytokine production yields were higher when using supernatants from the 40SA virus-infected cells than with IIIaSA, suggesting higher T-cell activation. As observed with luciferase, expressing FBiTE with 40SA induced higher transgene levels than with IIIaSA.

### 2.5. Generation and Characterization of Hyaluronidase-Expressing Oncolytic Adenoviruses

**Figure 3:**
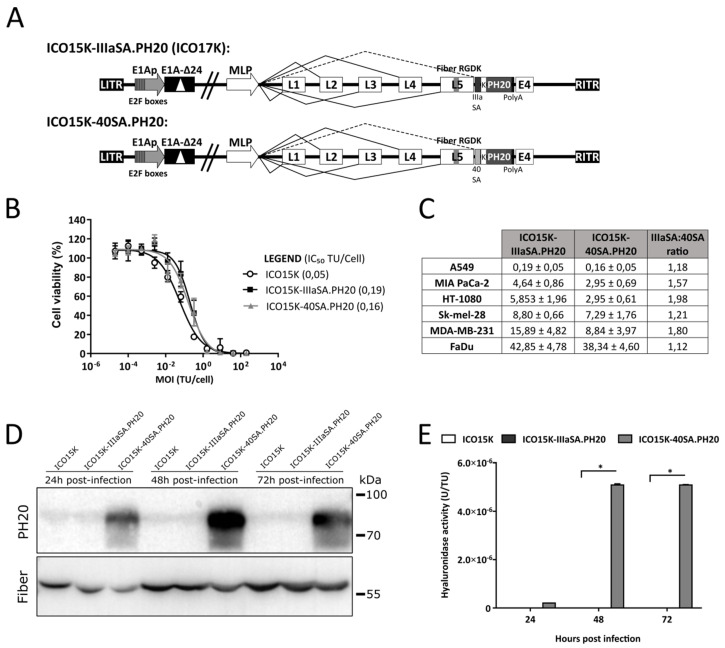
(**A**) Schematic representation of hyaluronidase-expressing OAds genomes. (**B**) Dose-dependent cytotoxic assay in A549 cells five days post-infection. (**C**) Table containing the IC_50_ values from cytotoxic assays in different cancer cell lines (four days post-infection). (**D**) Western blot detected the PH20 protein (upper panel) and adenovirus 5 fiber (lower panel); raw data are presented in Figure S2. (**E**) Turbidimetric assay to detect the hyaluronidase activity of supernatants from A549-infected cells at different timepoints. The absorbance at 600 nm was measured, and a standard curve with recombinant PH20 was used to extrapolate the enzimatyc units (U) of supernatants. The U value was normalized by the intital transfectiung units (TU). * *p* < 0.05 significant vs. ICO15K based on Kruskal–Wallis and Dunn’s *post hoc* tests.

Observing that 40SA offered higher transgene expression at the expense of virus replication using luciferase and FBiTE, we decided to test another therapeutic transgene, a soluble version of human sperm hyaluronidase (PH20; *SPAM1*). We published an ICO15K armed with PH20 After-fiber by IIIaSA transcription control, ICO15K-IIIaSA.PH20 (also known as ICO17K or VCN-01) [22]. Aiming to compare the SAs, we then generated ICO15K-40SA.PH20 (Figure 3A).

First, the cytotoxic properties of ICO15K-IIIaSA.PH20 and ICO15K-40SA.PH20 were evaluated in vitro in A549 cells. As previously published, the ICO15K-IIIaSA.PH20 virus did not lose its oncolytic potency compared to the parental ICO15K [22]. Surprisingly, no significant differences were found between IIIaSA and 40SA hyaluronidase-expressing viruses, contrary to our results with luciferase and FBiTE (Figure 3-B). To further evaluate this, a panel of cancer cell lines was used to screen the cytotoxic effect of both viruses. No consistent differences were observed except for HT-1080 and MD-MB-231 cell lines, where ICO15K-40SA.PH20 presented an almost 2-fold lower IC_50_, indicating more cytotoxicity, contrary to what could be expected from the luciferase and FBiTE results. No loss of oncolytic potency was observed in the 40SA-virus compared to the IIIaSA-virus (Figure 3C).

### 2.6. The 40SA Generated Higher Hyaluronidase Levels than IIIaSA

After the unexpected replication efficiency of ICO15K-40SA.PH20 at the same levels as ICO15K-IIIaSA.PH20, we decided to check the transgene expression kinetics. Supernatants from infected cells were assessed by Western blot against PH20 and adenovirus 5 fiber. PH20 was only detected in ICO15K-40SA.PH20 supernatants at every time point (24, 48, and 72 h post-infection). Conversely, no bands were specifically detected in ICO15K and ICO15K-IIIaSA.PH20 (Figure 3D, uncropped image in Supplementary Figure S2). Nevertheless, we did not observe significant differences in fiber levels between viruses at later times, which confirms the virus replication and enhanced PH20 expression associated with the 40SA.

The activity of the transgene was assessed in a turbidimetric assay. The supernatants were incubated overnight with hyaluronic acid to test their capacity to degrade it. Only ICO15K-40SA.PH20 supernatants significantly degraded hyaluronic acid (Figure 3E). Only upon concentrating the supernatants did we detect hyaluronidase activity by ICO15K-IIIaSA.PH20, as published (Supplementary Figure S3) [22]. Thus, 40SA significantly enhanced PH20 expression compared to IIIaSA, as observed with luciferase and FBiTE.

### 2.7. SA drives Oncolytic Acctivity in GC3-Rich Transgenes

According to our results, the viral fitness when arming After-fiber with SAs depends on the transgene. We studied three different transgenes without direct cytotoxic activity: luciferase, FBiTE, and PH20. The first two considerably affected viral replication, whereas PH20 did not hamper viral cytotoxicity. Moreover, arming with 40SA increased the loss of virus oncolytic potency compared with IIIaSA in luciferase- and FBiTE-armed OAds. In contrast, ICO15K-40SA.PH20 had similar IC_50_ values as ICO15K-IIIaSA.PH20. Aiming to understand these differences between transgenes, we analyzed the particular set of codons used by each transgene, also known as codon usage. Transgene codon usage has been recently described as one of the major drivers of OAd therapeutic efficacy when expressed by late units, where transgenes with extensive G or C at the third codon position (GC3) lead to oncolytic activity impairment [25]. Aiming to understand the differences between luciferase, FBiTE, and PH20, we analyzed the codons used by each transgene. Two different patterns were detected in the GC3 content analysis: luciferase and FBiTE presented high levels (57.1% and 59.2%, repectively) of GC3 codons, whereas the PH20 GC3 content was 35.6%.

**Figure 4:**
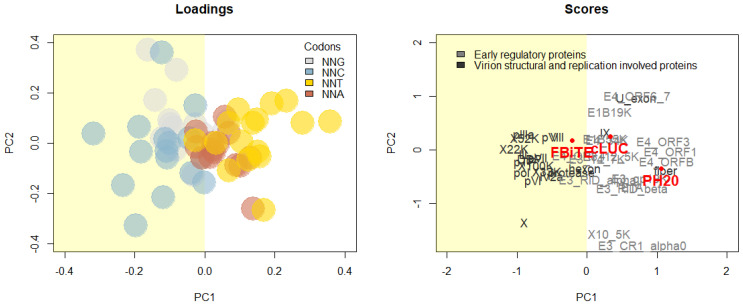
Transgene codon usage evaluation by Principal Component Analysis (PCA). Loadings in the left panel show codons colored according to the 3rd nucleotide; scores in the right panel show the distribution of viral genes, FBiTE, Luciferase, and PH20 transgenes along the first two principal components (PC1 and PC2). Genes with negative values for PC1 display GC3 biased codon usage. Genes with positive values for PC1 are related to higher use of AT3 codons.

A codon usage principal component analysis (PCA) was performed with Ad genes and the three transgenes tested in this study. According to their GC3 content, luciferase and FBiTE clustered together with late structural Ad genes, whereas the PH20 clustered with early regulatory genes (Figure 4, Scores). These results suggest that FBiTE and luciferase are more prone to compete with late genes for cellular translational resources; thus, overexpression of these transgene transcripts by 40SA further aggravates the interference with viral fitness. However, the AT3-biased codon usage of PH20 avoids the competition, allowing strong transgene expression by 40SA without interfering with viral oncolytic activity.

## 3. Discussion

Arming OAds has been one of the main strategies to enhance their antitumor efficacy. Different features should be considered to design armed OAds. The most important is the transgene, but the insertion site and its transcriptional control are crucial for successful therapy. Several insertion sites have been reported, most of them in the early 2000s. Herminston and co-workers described different insertion sites in partially or complete E3-deleted Ads [18,26,27,28,29]. Based on these studies, most armed OAds have deletions in E3. However, E3 proteins are involved in immune evasion, and their deletion reduces virus persistence in immunocompetent models in vivo [10,11,12] and probably in patients.

Therefore, non-E3 deleted viruses could longer sustain transgene expression. However, arming these OAds faces the challenge of an adenoviral packaging limit (2 kb over the wild type size). In terms of early or late transgene expression, it was reported that insertions driven by the major late promoter (MLP) confer higher expression than E3 promoters [20]. The reported insertion sites in non-E3 deleted viruses driven by the MLP are downstream of L3 [20] and downstream of L5 (After-fiber), generating a new transcription unit, also known as L6 [14,16]. In this study, we compared the After-fiber insertion site (proximal site) against the After-E4 insertion site (distal site), which has been described only in E3-deleted viruses [18,19].

We generated four different viruses expressing Click Beetle Green luciferase (Luc) in these two locations with two different splice acceptors. Our results confirmed that the distal transgene location between E4 and RITR offers significantly lower transgene levels than After-fiber (proximal to Major Late Promoter). The lower potency of the distal location was previously suggested by Fernández-Ulibarri et al. [19]. Although no comparisons armed with the same splice acceptor in these two different locations were reported, the authors obtained significantly lower levels in After-E4 armed OAd than in After-fiber. Here, we describe in vitro a head to head comparison, where After-fiber conferred more than a 200-fold higher expression than After-E4 at 72 h post-infection. On the other hand, this enhanced transgene expression was at the expense of oncolytic potency. ICO15K-E4-IIIaSA.Luc and ICO15K-E4-40SA.Luc replicated significantly better than their After-fiber-armed counterparts. According to this, we recommend the After-fiber insertion site for an enhanced transgene expression compared to the After-E4 transgene location. The latter may be better when a lower transgene expression is desired, for example, for transgenes that may interfere with virus fitness.

Apart from the insertion site, transgene transcriptional control is a key element when arming OAds. Autonomous transgene cassettes with exogenous promoters such as the cytomegalovirus (CMV) promoter have been widely used. Nevertheless, they are large DNA sequences and would drive transgene expression without tumor-selective restriction. Two strategies have been proposed to overcome this limitation: to substitute a virus gene with the transgene, taking advantage of endogenous promoters, or linking the transgene expression to the Ad gene. The first requires a deletion in the Ad genome, commonly partial E3 gene replacements. Late genes are indispensable for virus replication and cannot be replaced. Moreover, Ad early genes are expressed at lower levels and as a consequence, the transgene would be expressed at lower levels than late genes. The second strategy is to link the transgene to a virus gene using an internal ribosome entry site (IRES), a self-cleavable 2A viral peptide, or splice acceptors (SA). IRES are large DNA sequences (300–600 bp), and 2A viral peptide linkers reduce the viral replication yields [13,30]. Therefore, the use of SA (~50bp) is the most suitable approach to insert a transgene, creating a new reading frame in the Ad genome under the MLP by alternative splicing. Four different SA have been described in the literature [14,15,17,18].

In this study, we compared the Ad5 protein IIIa SA (IIIaSA) against the Ad40 long fiber SA (40SA). The 40SA produces significantly higher levels of expression compared to the IIIaSA. This is shown with three different transgenes: luciferase, FBiTE, and the PH20 transgene. ICO15K-40SA.Luc induced approximately 200-fold higher luminescence in vitro and in vivo than ICO15K-IIIaSA.Luc over time. ICO15K-40SA.FBiTE secreted higher amounts of FBiTE molecules than ICO15K-IIIaSA.FBiTE as suggested by the binding experiments and T-cell activation assays. Finally, the PH20 protein and activity were only detected in supernatants from ICO15K-40SA.PH20-infected cells. The PH20 detection in ICO15K-IIIaSA.PH20 supernatants required a 20-fold concentration, confirming a higher transgene production of 40SA than IIIaSA.

Consistent results were obtained with the three different transgenes in terms of their expression: proximal After-fiber location and 40SA allowed more expression than After-Fiber IIIaSA and distal E4 location. Nonetheless, OAds armed with luciferase or FBiTE lost cytotoxic potency compared to the parental virus, and the enhanced transgene expression driven by 40SA magnified this interference with virus fitness. Surprisingly, this effect was not observed with PH20, where ICO15K-40SA.PH20 strongly expressed higher PH20 levels than the IIIaSA-virus, without any loss in virus replication. We studied the different features of these transgenes. Luciferase, FBiTE, and PH20 have similar DNA lengths, all of them under 2 kb, discarding the adenovirus packaging limitation. Luciferase is intracellular, while FBiTE and PH20 are secreted proteins, but they do not have cytotoxic properties by themselves. In line with previous observations by our group, we hypothesized that the transgene sequence might influence virus replication of armed OAds [25]. We studied the particular set of codons used for these transgenes, also known as codon usage. Luciferase and FBiTE presented a codon usage rich in codons with G or C at the third base position (related to adenovirus oncolytic activity impairment). By contrast, PH20 showed a codon usage biased toward codons with A or T at the third base position and poor in GC3 codons (described as compatible with efficient adenoviral oncolytic activity).

Achieving high expression of the therapeutic transgenes without reducing virus oncolytic potency is one of the major challenges of oncolytic virotherapy. Traditionally, codon usage optimization (increasing the GC3 sequence content) has been used to increase transgene protein yields. However, this strategy, broadly used in gene therapy, is not suitable in the oncolytic virotherapy context, as excessively optimized transgenes are susceptible to trigger competition for cellular translational resources with viral genes, leading to virus fitness impairment. Our study contributes to reinforce this idea that late transgenes with a high GC3 content lead to virus impairment.

Our results are in line with the concept of virus-transgene competition for translational cellular resources as we demonstrated that virus impairment is favored not only by the transgene GC3 content, but also by any mechanism that increases transcription of transgenes with a similar codon usage to the late viral genes [31]. In this context, our results suggest that it is possible to overcome transgene high expression and viral fitness incompatibility by arming transgenes with a poor GC3 content but controlled under a strong SA, such as 40SA. Despite the need for more comprehensive studies to characterize the interplay between the expression of transgenes and virus genes, our findings represent a new step forward to improve the design of armed OAds.

## 4. Materials and Methods 

### 4.1. Cell Lines

Human cell lines A549 (lung adenocarcinoma), 293 (embryonic kidney), HT-1080 (fibrosarcoma), Jurkat (T-cell leukemia), MIA PaCa-2 (pancreatic carcinoma), Sk-mel-28 (melanoma), MDA-MB-231 (breast adenocarcinoma), and FaDu (pharynx squamous cell carcinoma) were obtained from the American Type Culture Collection (ATCC). The 293mFAP and 293hFAP cell lines were kindly provided by Dr. Eric Tran (National Institutes of Health, Bethesda, MD, USA). HT-1080 FAP-expressing cell lines were generated by transducing a lentivirus encoding either murine or human FAP cDNA (Dharmacon, Lafayette, CO, USA). They were sorted and expanded. Cells stably expressing murine FAP or human FAP were designated as HT-mFAP or HT-hFAP, respectively. All tumor cell lines were maintained in Dulbecco’s modified Eagle’s medium (DMEM) supplemented with 10% fetal bovine serum (FBS, Invitrogen, Carlsbad, CA, USA) and 1x Penicillin/Streptomycin (PS, ThermoFisher Scientific, Waltham, MA, USA) except for Jurkat cells, which were cultured with RPMI-1640 medium at 10% FBS and 1% PS. All cells were maintained in a 37 °C, 5% CO_2_ incubator, and were routinely tested for mycoplasma.

### 4.2. Isolation and Expansion of T Cells

All experiments were approved by the ethics committees of the University Hospital of Bellvitge and the Blood and Tissue Bank (BST) from Catalonia (project 140015, approved in 22/05/2014). Blood samples were obtained from the BST. Peripheral blood mononuclear cells (PBMCs) were isolated by ficoll density gradient centrifugation. PBMCs were treated with ACK lysis buffer (Lonza, Switzerland) and resuspended in RPMI-1640 medium supplemented with 10% FBS. T cells were isolated using the Rossette-Sep Human T-cell enrichment cocktail (STEMCELL Technologies, France). For stimulation, T cells were cultured with CD3/CD28-activating Dynabeads (ThermoFisher Scientific, Waltham, MA, USA) at a 1:3 bead-to-cell ratio. Cells were counted and fed every day until day 10, when they were either used for functional assays or cryopreserved.

### 4.3. Construction of Recombinant Adenoviruses

We used the ICO15K as a platform for introducing transgenes. ICO15K was previously described [23]. Sequences of splice acceptors, insertion site homologies, and primers to generate transgenes by PCR are detailed in Supplementary Tables S1 and S2. Click Beetle Green luciferase (Luc) was introduced downstream of the fiber gene or between E4 and RITR under IIIaSA [15] and 40SA [17] splice acceptors by homologous recombination in bacteria [32,33]. The FBiTE transgene and ICO15K-IIIaSA.FBiTE were previously described and generated [21]. We replaced the IIIaSA with 40SA in ICO15K-40SA.FBiTE by homologous recombination. ICO15K-IIIaSA.PH20, also known as ICO17K or VCN-01, was previously published [22]. IIIaSA was substituted for 40SA generating ICO15K-40SA.PH20 by homologous recombination. Newly generated adenoviral genomes (luciferase-expressing viruses, ICO15K.40SA-FBiTE, and ICO15K-40SA.PH20) were transfected in 293 cells using calcium phosphate. Viruses were rescued post-transfection. The individual clone of each virus was selected by plaque assay and was propagated in A549 cells. Viruses were purified by two sequential cesium chloride gradients and dialysis. Functional (transducing units (TU)/mL) and physical (viral particles (vp)/mL) titers of purified viruses were determined by anti-hexon [34] staining and optical absorbance at 260 nm, respectively.

### 4.4. Virus Cytotoxicity Assays

Virus cytotoxicity assays were performed as previously described [23]. Briefly, a serial dilution of viral transfecting units (TU) was used to infect the desired cell line in triplicate. The initial multiplicity of infection (MOI; TU/cell) and the number of cells were adjusted according to Table 1 depending on the cell line. After 4–5 days of infection, the cell viability was assessed by bicinchoninic acid assay (BCA, Pierce Biotechnology). Absorbance was quantified, and the number of TU per cell required to produce 50% inhibition (IC_50_) was estimated from a dose-response non-linear regression with a variable slope, calculated with GraphPad Prism v6.02 (GraphPad Software Inc.,San Diego, CA, USA).

### 4.5. Luciferase Reporter Assays

A549 cells (3 × 10^4^ cells/well) were infected at an MOI of 0,5 in 200 µL with ICO15K-IIIaSA.Luc, ICO15K-40SA.Luc, ICO15K-E4-IIIaSA.Luc or ICO15K-E4-40SA.Luc in 96-well plates. After 24, 48, and 72 h of incubation, the medium was collected and centrifuged and supernatants were discarded. Then, 50 µL of Reporter Lysis Buffer (Promega, Madison, WI, USA) was added to the pellets, mixed, and added to the cells in 96-well plates. After freezing the plates for 24 h, 10 µL of cell lysate and 25 µL of luciferase Assay Reagent (LAR, Promega) were added to 96-well white plates. The emitted luminescence was analyzed on a luminescence plate reader (Wallac 1420 Victor multilabel counter, PerkinElmer, MA, USA).

The in vivo study was performed at the Biomedical Research Institute of Bellvitge (IDIBELL) facility (AAALAC unit 1155) and was approved by the Ethics Committee for Animal experimentation from IDIBELL. Six-week-old female SCID/Beige mice (Envigo) were subcutaneously implanted with 4 × 10^6^ A549 cells in both flanks. Once tumors reached a median volume of 120 mm^3^, mice were randomized and intratumorally treated with 1 × 10^9^ vp/tumor. Mice were monitored over time, and luciferase expression was assessed by injecting 200 µL of D-luciferin potassium solution (15 mg/mL, Byosinth AG) intraperitoneally and imaged using an IVIS Lumina XRMS Imaging system (PerkinElmer, MA, USA). Tumor luminescence was measured by drawing a region of interest (ROI) around the tumor contour.

### 4.6. Production of Supernatants

A549 cells were seeded at 1 × 10^7^ cells in 10 mm culture plates. When plates reached 90% confluence, the medium was removed, and cells were infected with the desired virus at 20 TU/cell in a final volume of 10mL of DMEM 5% FBS. At 72 h post-infection, supernatants were harvested and centrifuged for 5 min at 500 g to spin down the detached cells. Supernatants from uninfected cells were used as a mock control. For binding assays (see 4.7), supernatants were concentrated (20×) with Amicon Ultra-15 filter units with a molecular weight cutoff of 30 kDa (Merck Millipore, MA, USA) according to the manufacturer’s protocol.

### 4.7. Binding Assays

Tumor cell lines (2 × 10^5^ cells) or Jurkat cells (1 × 10^5^) were incubated with the desired supernatant for 1h on ice. Then, cells were washed thrice with PBS+2% BSA followed by an incubation for 30 min on ice with monoclonal M2 anti-FLAG tag antibody (Sigma Aldrich, Bozeman, MO, USA) or the corresponding IgG1 isotype control (Santa Cruz Biotechnology, Dallas, TX, USA). Cells were washed again (three times) and incubated for 30 min in the dark on ice with secondary goat anti-mouse IgG coupled with Alexa Fluor 488 (Thermo Fisher Scientific, Waltham, MA, USA). Flow cytometry analysis was performed on a Gallios cytometer (Beckman Coulter, CA, USA), and data were processed with FlowJo v7.6.5 (Tree Star, Daytona Beach, FL, USA).

### 4.8. T-cell Activation Assay

Tumor cells (3 × 10^4^ cells) and T cells (effector-to-target ratio of 5) were seeded in 96-well plates in 100 µL of medium, and 100 µL of the supernatants were added to the wells. Supernatants were collected after 24 h of incubation and assessed for human IFN-ɣ, TNF-α, and IL-2 using the ELISA MAX Deluxe set (Biolegend, San Diego, CA, USA), following the manufacturer’s protocol.

### 4.9. Western Blot

Supernatants from A549 infected cells with ICO15K, ICO15K-IIIaSA.PH20, and ICO15K-40SAPH20 at 20 TUs/cell were harvested 24, 48, and 72 h post-infection. Supernatants were resolved by electrophoresis on an 8% acrylamide gel and transferred to a nitrocellulose membrane by standard methods. Then, membranes were immunoblotted with anti-PH20 antibody (Novusbio, ref. NBP1-81637) for hyaluronidase detection and with anti-Adenovirus 5 fiber antibody (Fitzgerald, ref. 10R-A116B) for fiber detection. Membranes were incubated overnight at 4 °C and secondary labeled with correspondent anti-mouse (DAKO, ref. P0447) and anti-rabbit antibodies (DAKO, ref. P-0448) according to the manufacturer’s protocol. Bands intensity were quantified by Image Lab v6.0.1 (Bio-Rad Laboratories, Inc.).

### 4.10. Assay for Hyaluronidase Activity—Turbidimetric Assay

A549 cells were infected with 20 TU/cell with ICO15K, ICO15K-IIIaSA.PH20, and ICO15K-40SA.PH20. Four hours after infection, cells were washed with PBS, and we added a fresh medium. Supernatants were harvested at 24, 48, and 72 h. To assess their hyaluronidase activity, they were mixed with hyaluronic acid (HA, Sigma Aldrich, Burlington, MA, USA) solution in phosphate buffer (pH = 5.35) and incubated overnight at 37 °C. Then, the reaction wass stopped by adding 5 volumes of acid albumin solution (24mM sodium acetate, 79 mM acetic acid, and 0.1% of bovine albumin (pH = 3.75)), and the absorbance at 600 nm was measured. Low absorbances indicate high hyaluronidase activity. A blank control containing fresh medium and HA solution and a positive control with recombinant PH20 (PH0-H5225, Acro Biosystems, Newark, DE, USA) were included.

### 4.11. Codon Usage Analysis

The codon usage analysis was performed as previously published [25]. Briefly, the codon usage frequencies were analyzed using the Sequence Manipulation Suite. The relative codon usage was calculated normalizing the codon usage of each codon by every synonymous codon for each amino acid. Relative codon usage was used to perform the Principal Component Analysis (PCA) with R v3.2.3 software.

### 4.12. Statistical Analysis

Statistical analyses were performed using GraphPad Prism software v6.02 (GraphPad Software Inc., San Diego, CA, USA) All results were expressed as means ± SD or SEM, as indicated. A normality test was performed, and a corresponding parametric or non-parametric test was used. For parametric samples, a two-tailed unpaired Student’s T-test was used to evaluate the statistical significance between two groups. One-way ANOVA with Tukey’s *post hoc* test was used for differences between three or more groups for a single condition or time point. Two-way ANOVA with Tukey’s *post hoc* test was used for comparisons of three or more groups over time. For non-parametric samples, Kruskal–Wallis and Dunn’s *post hoc* tests were used to compare more than two groups and non-repeated measures. *p* < 0.05 was taken as the level of significance.

## 5. Conclusions

After-fiber transgene location confers higher transgene expression than After-E4 insertion site when arming with splice acceptors. A direct comparison between 40SA and IIIaSA indicates that the former leads to higher expression. Transgene codon usage is important in terms of virus fitness. Transgenes expressed by the major late promoter with similar codon usage as late/structural adenoviral genes (except fiber) reduce virus replication and when overexpressed critically impact virus fitness. On the other hand, transgenes expressed with a codon usage different than the adenoviral structural genes or similar to the fiber will not reduce oncolytic potency. We suggest that using transgenes with a low GC3 content under the 40SA inserted downstream of the fiber gene results in the optimal design to achieve high expression with efficient virus replication.

## Figures and Tables

**Table 1:** Detailed number of cells and intial multiplicity of infection (MOI) for in vitro cytotoxic assays.

	Cells Per Well	Initial MOI (Serial Dilution)
A549	30,000	200 (1/5)
Sk-mel-28	20,000	200 (1/5)
MIA PaCa-2	30,000	200 (1/3)
FaDU	20,000	600 (1/3)
HT-1080	20,000	600 (1/3)
MDA-MB-231	15,000	400 (1/3)

## Supplementary Materials

The following are available online at https://www.mdpi.com/1422-0067/21/14/5158/s1, Figure S1: Schematic representation of Adwt and ICO15K, Figure S2: Uncropped anti-PH20 Western Blot, Figure S3: Hyaluronidase activity of concentrated supernatants from ICO15K, ICO15K-IIIaSA.PH20, and ICO15K-40SA.PH20, Table S1: Detailed sequences of SAs and insertion sites, Table S2: Primers used for homologous recombination.
