# Supplementary material for: Arming Oncolytic Adenoviruses: Effect of Insertion Site and Splice Acceptor on Transgene Expression and Viral Fitness
Source: Int J Mol Sci. 2020 Jul 21;21(14):5158. doi: 10.3390/ijms21145158 (PMC7404292; doi:10.3390/ijms21145158)
Supplement: Supplementary file 1 [file ijms-21-05158-s001.pdf]

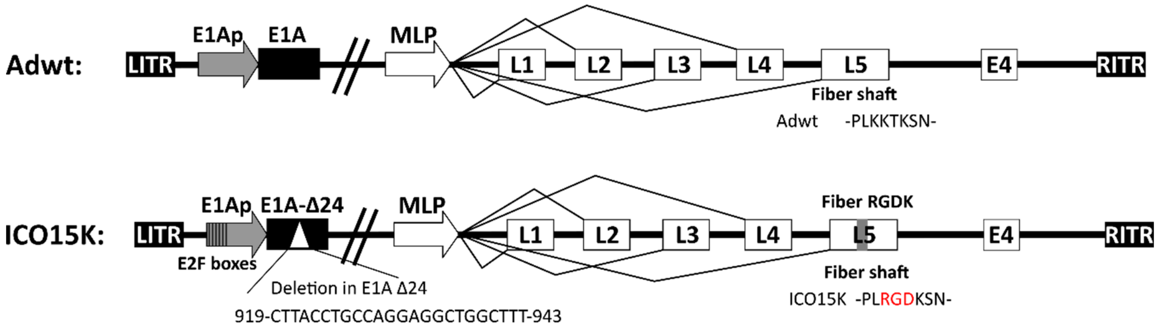

Figure S1. Schematic representation of Adenovirus serotype 5 (Adwt) and ICOVIR-15K (ICO15K) genomes.

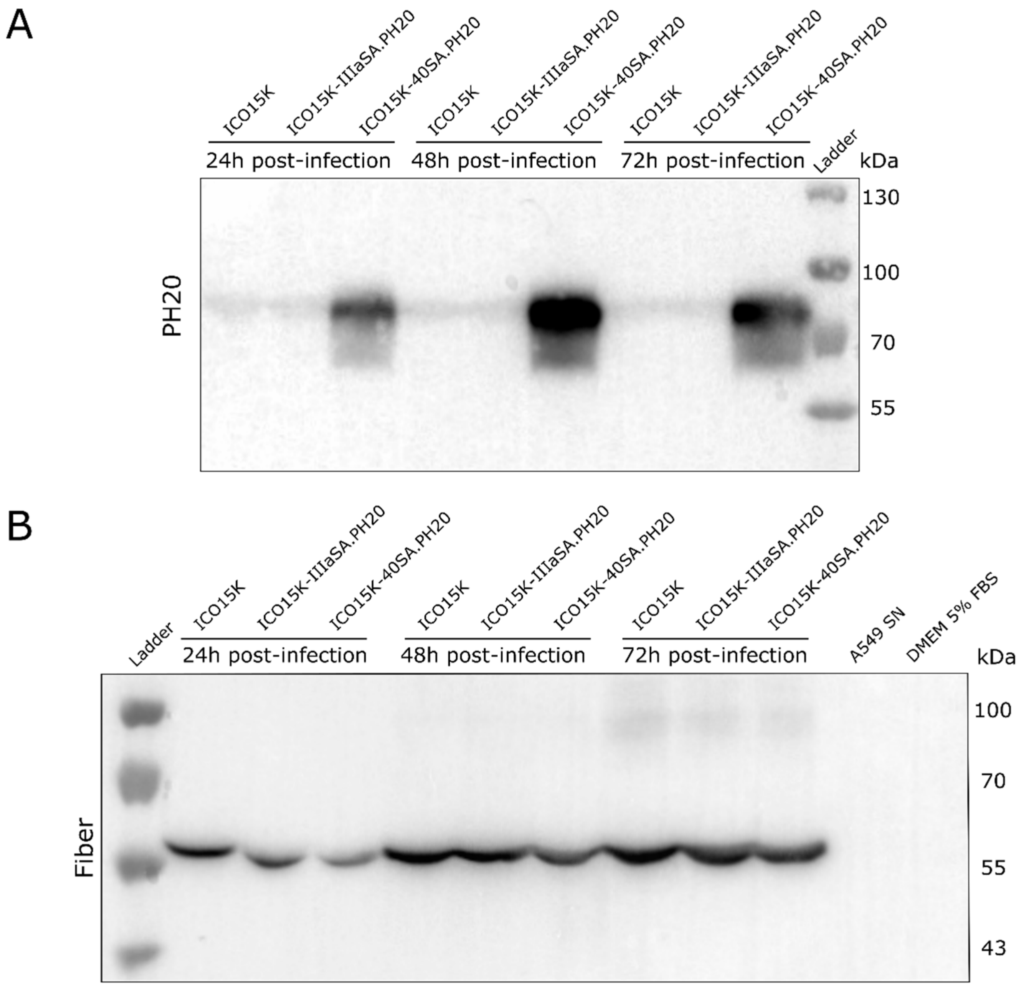

C

	24h post-infection			48h post-infection			72h post-infection				
	ICO15K	ICO15K-IIIaSA.PH20	ICO15K-40SA.PH20	ICO15K	ICO15K-IIIaSA.PH20	ICO15K-40SA.PH20	ICO15K	ICO15K-IIIaSA.PH20	ICO15K-40SA.PH20	A549 SN	DMEM 5%FBS
**PH20**	1847600	1505280	12930048	2083146	2289567	28114905	1947540	1627438	19214760	NA	NA
**Fiber**	9325329	6748920	5034479	13057762	12820440	11249520	15009666	13513071	12918906	Undetected	Undetected
**Ratio**	0,19812706	0,22304013	2,568299123	0,159533157	0,17858724	2,4992093	0,12975239	0,12043436	1,48733647	NA	NA

Figure S2. (**A**) Uncropped membrane of Western blot against human PH20. (**B**) Uncropped membrane of Western blot against Adenovirus serotype 5 fiber. (**C**) Table containing the densitometry of western blot bands and the PH20:Fiber ratios.

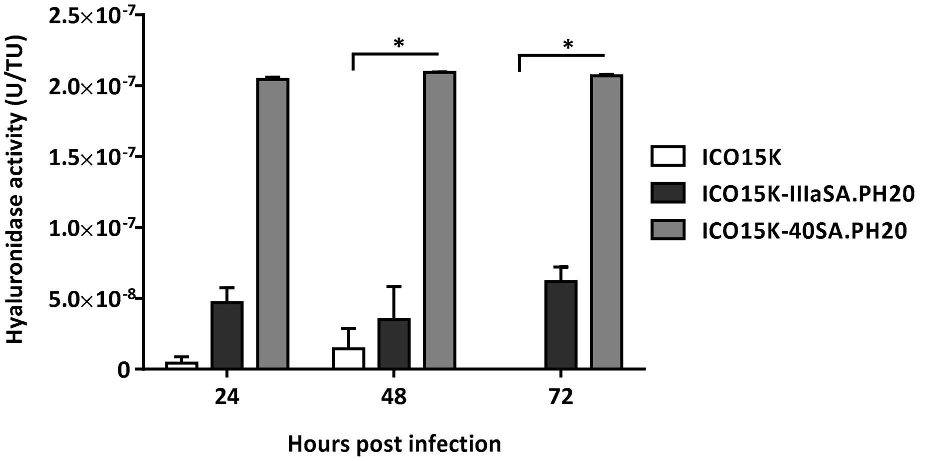

Figure S3. Hyaluronidase activity of concentrated supernatants from infected A549 at different time points. A549 cells were infected with the indicated viruses at MOI 20. Supernatants were harvested at 24, 48, or 72hours post-infection and 20x concentrated with AMICON (as detailed in Materials and Methods, 4.6). Hyaluronidase activity was measured by turbidimetric assay and normalized for the initial transfecting units (TU). The ICO15K-40SA.PH20 reached saturation levels in the assay impeding an exact quantification of hyaluronidase activity (all hyaluronic acid was degraded). *$p<0.05$ significant by Kruskal-Wallis test and Dunn's *post hoc* test.

Supplementary Table 1. Detailed sequences of splice acceptors, kozak sequence, polyA signal, and insertion sites used in this study.

Name	Observations	Sequence
IIIaSA	Splice acceptor Ad5 IIIa gene	5’gtactaagcggtgatgtttctgatcag 3’
40SA	Splice acceptor Ad40 long fiber gene	5’gcaggcgcaatcttcgcatttctttttccag 3’
Kozak sequence	Translation initiation signal	5’ ccacc 3’
PolyA sequence	Minimal polyA signal	5’ aataaa 3’
After Fiber homology upstream	Insertion site After fiber, upstream homology with Ad5	5’ctcttacactttttcatacattgcccaagaataaagaatcgtttgtgttatgtttcaacgtgtttatttttcaattg 3’
After Fiber homology downstream	Insertion site After fiber, downstream homology with Ad5	5’ctttatttttcaattgcagaaaatttcaagtcatttttcattcagtagtatagccccaccaccacatagcttataca 3’
After-E4 homology upstream	Insertion site After E4, upstream homology	5’ccaaaaaacccacaacttcctcaaatcgtcacttccgttttcccacgttacgtcac 3’
After-E4 homology downstream	Insertion site After E4, downstream homology	5’cattttaagaaaactacaattcccaacacatacaagttactc 3’

Supplementary Table 2. Primers used to generate transgenes by PCR for subsequent homologous recombination.

Virus	Primers	Template
ICO15K-IIIaSA.Luc	Fwd:5’**caattgg***gtactaagcggtgatgtttctgatcag*ccaccatggtaaagcgtgagaaaaatgt 3’<br>Rv:5’**tgacttgaaattttctgcaattgaaaaataaag**tttattaaccgccggccttctc 3’	pTRPE CBG T2A GFP
ICO15K-40SA.Luc	Fwd:5’**cgtttgtgttatgtttcaacgtgtttatttttcaattggcaggcgcaatcttg***gcatttctttttccag*ccaccatggtaaagcgtgagaaaaatgt 3’<br>Rv:5’**tgacttgaaattttctgcaattgaaaaataaag**tttattaaccgccggccttctc3’	pTRPE-CBG-T2A-GFP
ICO15K-E4-IIIaSA.Luc	Fwd:5’**ccaaaaaacccacaacttcctcaaatcgtcacttccgttttcccacgttacgtcac***gtactaagcggtgatgtttctga* 3’<br>Rv:5’**gtaggttttagggcggagtaacttgtatgtgttgggaattgtagttttcttaaaatg**tttattaaccgccggccttctccaaca 3’	ICO15K-IIIaSA.Luc
ICO15K-E4-40SA.Luc	**Fwd:5’ccaaaaaacccacaacttcctcaaatcgtcacttccgttttcccacgttacgtcac***gcaggcgcaatcttcgcatt* 3’<br>Rv:5’**gtaggttttagggcggagtaacttgtatgtgttgggaattgtagttttcttaaaatg**tttattaaccgccggccttctccaaca 3’	ICO15K-40SA.Luc
ICO15K-40SA.FBiTE	Fwd:5’**atcgtttgtgttatgtttcaacgtgtttatttttcaattg***gcaggcgcaatcttcgcatttctttttccag*ccaccatgggatggtcctg 3’<br>Rv:5’**gctatactactgaatgaaaaatgacttgaaattttctgcaattgaaaaataaag**tttattacttgtcgtcgtcgtcc 3’	ICO15K-IIIaSA.FBiTE
ICO15K-40SA.PH20	Fwd:5’**atcgtttgtgttatgtttcaacgtgtttatttttcaattg***gcaggcgcaatcttcgcatttctttttccag*ccaccatgggagtgctaaaattcaa 3’<br>Rv:5’**gctatactactgaatgaaaaatgacttgaaattttctgcaattgaaaaataaag**ttta**tta**agatagtgtggagggtg 3’	ICO15K-IIIaSA.PH20
**Bold:** homology with Ad5, *Italic:* correspondent SA, Underlined: Kozak sequence (Forward) or stop codon+PolyA (Reverse)		
